# Perceptions About Aging and Aesthetics: A Global Study of Adults Aged 50 to 80 Years

**DOI:** 10.1093/asjof/ojaf127

**Published:** 2025-11-13

**Authors:** Stephanie Manson Brown, Shannon Humphrey, Carolina Reato Marçon, Cristiane Benvenuto-Andrade, Sylwia Lipko-Godlewska, Ada Trindade de Almeida, José Raύl Montes

## Abstract

**Background:**

Aesthetic treatments are associated with positive psychological and psychosocial outcomes, but most data are from adults <60 years. As the global population ages, there is an opportunity to better understand perceptions about aging among older adults and whether engaging with aesthetic medicine influences these perceptions.

**Objectives:**

This global survey sought to understand the psychology of aging among older adults with varying relationships to aesthetic medicine.

**Methods:**

This online survey (July-September 2022) queried adults (50-80 years) in 8 countries. Respondents were divided into 3 groups: those that had ever received aesthetic treatments (aesthetics receivers); those naive to aesthetic medicine but considering (naive considerers); and those naive and not considering (naive non-considerers). Respondents were queried about perceptions and expectations of aging and perceptions of aesthetic treatments.

**Results:**

Among 7588 total respondents, 39.8% were aesthetic receivers, 28.7% were naive considerers, and 31.5% were naive non-considerers. Overall, satisfaction with psychological self-perceptions (eg, level of self-confidence) was high (∼80%) regardless of respondents' engagement with aesthetic medicine. Naive non-considerers had the most positive view on aging, but there was strong agreement across all groups that aging was associated with benefits (eg, more time for hobbies/leisure) and potential challenges (eg, changes in mobility, losing independence). Most respondents (83%), regardless of their relationship with aesthetic medicine, agreed that aesthetic treatments had emotional benefits (eg, feeling like the best version of oneself).

**Conclusions:**

The data from this multi-country survey of older adults gives key insights into perceptions of aging and aesthetic medicine among a previously underrepresented population.

**Level of Evidence: 5:**

(Therapeutic) 
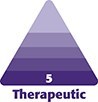

Aging is a multifactorial process that affects both physiological and psychological function. Globally, there is a rapid shift in the population toward older ages. The overall global life expectancy is expected to increase to 78.1 years by 2050, and healthy life expectancy (ie, the number of years it is estimated that a person can anticipate living in good health) is expected to increase to 67.4 years by 2050.^1^ As a result, the United Nations projects that by 2050, the number of adults aged 60 years or older worldwide will pass 1.5 billion, and the number of adults over 80 years old will surpass 450 million.^2^

The aging process is inevitable and allows for the integration of life's multiple and rich experiences; but can we age *better*? Data increasingly demonstrate a bidirectional relationship between an individual's outlook on aging and their overall health. Indeed, positive perceptions of aging and a positive outlook on life are associated with positive health outcomes and reduced mortality.^3-6^ The opposite relationship also exists, with an older perceived age being a risk factor for depressive symptoms and physical morbidity.^7,8^ Older adults with more negative self-perceptions related to aging (ie, higher levels of perceived uselessness) also had increased mortality.^9,10^

In the 20th century, gerontology tended to differentiate older adults largely based on the presence or absence of pathology. In the absence of disease, age itself was viewed as deterministic for physiological and cognitive changes, and age-related changes (eg, decreases in bone density, increased blood pressure, and mild memory impairment) were viewed as “normal” without accompanying risk. The contributory effects of lifestyle and psychosocial factors on health were minimized, and the heterogeneity of older adults' health was not well captured. In an effort to improve conceptualizations and understanding of aging, the idea of “successful” aging was proposed.^11,12^ “Successful” aging is a framework that exists as an alternative to “usual” aging and comprises 3 factors: the absence or avoidance of disease and/or disability, sustained physical and cognitive health, and maintenance of social engagement.^11,13^ More specific factors thought to contribute to successful aging include independence and active engagement with life, physical health (eg, mobility, remaining physically active), resilience and acceptance (eg, outlook on life, self-acceptance), social-emotional connections, health promotion and maintenance (including emotional and mental well-being), and being cognitively sound.^13,14^ It is important to note that the specific elements of successful aging may differ across cultures depending on the relative value of familial relations, autonomy, independence, etc.^15^

Aesthetic medicine can be a tool to enhance successful aging. In a study that sought to identify factors that are associated with successful aging, physical appearance was noted as a component of successful aging by only a few participants (though exact participant numbers were not reported).^13^ However, multiple studies have demonstrated positive psychological and psychosocial effects of aesthetic treatments, and thus, physical appearance may impact some of the contributing factors to successful aging.^16,17^ Aesthetic treatments, both surgical and minimally or noninvasive methods, have been historically used as a means to minimize or slow physical manifestations of aging (eg, age-related changes in skin quality, facial volume). But attitudes surrounding aesthetic medicine are changing. Increasingly, aesthetic treatments may be viewed as a tool to feel empowerment in the aging process and align self-image with external appearance. Aesthetic medicine has evolved to include regenerative approaches to improve and maintain skin health or as a means of self-care (ie, a way to care for oneself), which contrasts with engaging with aesthetic medicine as a way to “fix” signs of aging. For older women, in particular, who often report feeling invisible, devalued, or underrepresented in multiple facets of society because of an intersection of ageism and sexism, aesthetic medicine, when used to boost confidence and authenticity, could foster successful aging.^18,19^ Additionally, there is increased acceptance and decreased stigmatization around receiving aesthetic treatments.

Most published literature within aesthetic medicine, and particularly publications of clinical trials, report data from participants under <60 years of age. This does not mean, however, that older patients are not seeking or actively engaging with aesthetic medicine but leaves the experiences and voices of older individuals underrepresented. As a result, there is an opportunity to understand the motivations of older adults for engaging, or not engaging, with aesthetic medicine.

Our goal of the present article is to better understand perceptions about aging and appearance among older adults (50-80 years) and how these perceptions are influenced by individuals' relationships with aesthetic medicine. Our aim is to give voice to an underrepresented and rapidly growing segment of the global population and explore how aesthetic medicine can better support how we age. Additionally, we want to explore what motivates older adults to seek aesthetic treatments.

## METHODS

### Survey Design and Respondent Eligibility

This online survey was conducted from July to September 2022. The survey, developed in consultation with a cognitive psychologist, queried respondents on their current perceptions and expectations of aging vs 10 years ago, and about perceptions of aesthetic treatments. All questions within the survey contained predetermined answer choices. Questions were presented in fixed order but answer options were presented in random order. Before beginning the survey, respondents consented to the collection of their responses for aggregate analysis and for use in publications in accordance with applicable data protection laws (ie, General Data Protection Regulation).

Prospective respondents were sourced from a general panel of consumers who had opted in to being contacted for online surveys (not limited to surveys about aesthetic medicine). Respondents interested in the survey provided their consent to participate before completing a preliminary set of questions to confirm eligibility, capture basic demographic characteristics, and divide participants into 3 subgroups based on their engagement with aesthetic medicine.

Eligible respondents were adults aged 50 to 80 years who live in Brazil, China, Germany, Israel, Japan, Spain, the United Kingdom, and the United States. Respondents were ineligible if they were diagnosed with Alzheimer's disease or other dementias; bipolar disorder; or psychotic disorders, including schizophrenia.

Respondents were divided into 3 subgroups based on their history of receiving aesthetic treatments and their willingness to receive aesthetic treatments in the future: those that had received aesthetic treatments (aesthetics receivers); those naive to aesthetics treatments but considering (naive considerers); and those naive to aesthetic treatments and not considering (naive non-considerers).

Responses were compared with 95% CIs and statistical significance was determined using *t*-tests. Results are summarized.

## RESULTS

### Respondent Demographics and Aesthetic Treatment History

A total of 7588 respondents (72.4% women, 27.6% men) completed the survey, with the largest number of participants from the United States (*n* = 2125), followed by China (*n* = 998), Spain (*n* = 900), Japan (*n* = 893), the United Kingdom (*n* = 827), Brazil (*n* = 825), Germany (*n* = 825), and Israel (*n* = 195). Respondents had a mean age of 65 years; 41% (*n* = 3125) were aged 50 to 59 years old, 37% (*n* = 2774) were aged 60 to 69 years old, and 22% (*n* = 1689) were 70 to 80 years old.

Among aesthetics receivers (*n* = 3017), 68% reported having ever received a facial (at a day spa, med-spa, or doctor's office), followed by 62% having ever received neurotoxin injections, 48% having ever received professional-grade skin care, 46% having ever received soft-tissue filler injections, and 46% having ever received cosmetic surgery. In the last 12 months, aesthetics receivers most frequently reported having received toxin injections (27%), facials (21%), professional-grade skin care (16%), and soft-tissue filler injections (15%). Respondents indicated that the treatments they were considering from a med-spa, aesthetician, or doctor in the next 12 months included cosmetic surgery (aesthetics receivers, 37%; naive considerers, 39%), neurotoxin injections (aesthetics receivers, 37%; naive considerers, 25%), and facials (aesthetics receivers, 31%; naive considerers, 29%).

### General Perceptions of Aging

**Figure 1.**
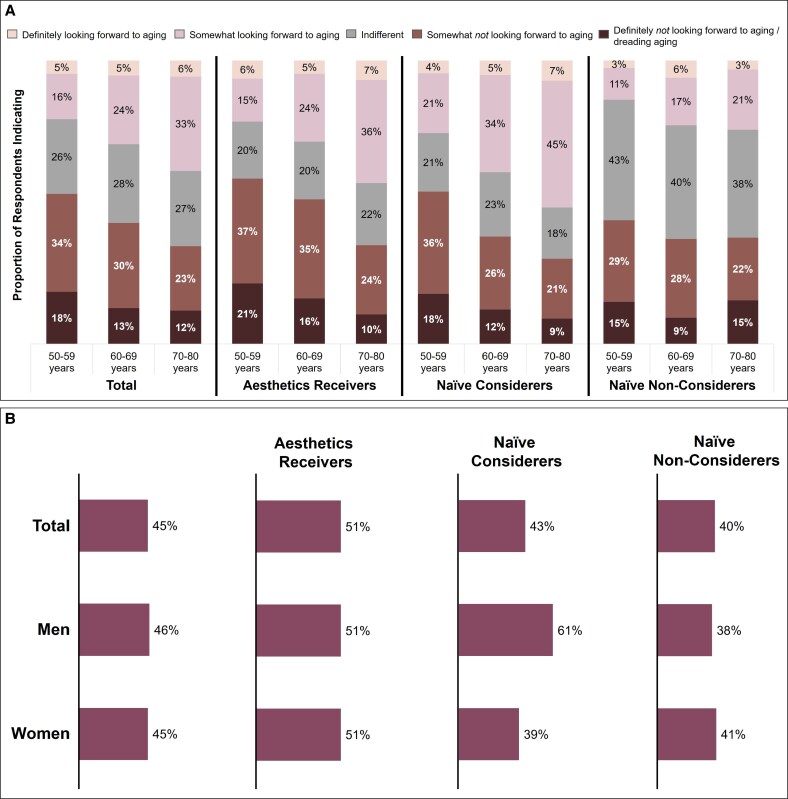
General perceptions on aging. Respondents were asked “Which of the following best matches your perceptions about getting older?” and given 5 answer options. (A) General perceptions of aging based on age subgroup and aesthetic engagement, expressed as a proportion of the respondents selecting each option. (B) Proportion of respondents who selected that they were “somewhat not looking forward to aging” or “definitely not looking forward to aging” based on self-identified gender and aesthetic engagement. Total, *N* = 7588. Men, *n* = 2092. Women, *n* = 5496.

Younger respondents (50-59 years) were more likely than either subgroup of older respondents (60-69 or 70-80 years) to report that they were *not* looking forward to aging (Figure 1). General perceptions of aging were similar between male and female respondents, although male naive considerers (61%) were more likely than female naive considerers (39%) to say they were not looking forward to aging. There were high levels of agreement between each respondent subgroup on the perceived benefits of getting older (Table 1), which include spending more time for hobbies/leisure (54%), more time with family and friends (53%), retirement (49%), and a slower pace of life (49%). Responses were similar across aesthetic engagement subgroups.

**Table 1.** Benefits Associated With Growing Older

Benefit	Total *N* = 7588, %	Aesthetics receivers *n* = 3017, %	Naive considerers *n* = 2180, %	Naive non-considerers *n* = 2391, %
More time for hobbies/leisure	54	55	52	53
More time to spend with family/friends	53	54	54	51
Retirement/not having to work	49	49	46	52
Slower pace of life/less stress	49	50	45	52
More time for travel	49	52	50	44
Wiser	46	45	45	46
More content	34	36	33	33
More financially secure	33	36	34	29
Increased confidence/more self-assured	33	36	35	28
Less pressure on how I should look	31	30	29	35
More discretionary income/fewer financial commitments	29	33	29	25
There are no benefits to getting older	9	8	7	11

Factors involved in aging that respondents were most concerned about included changes in physical health (66%), losing mobility (55%), changes in mental health and/or memory loss (54%), changes in appearance (45%), and losing family/friends (44%) (Table 2). Fears related to the aging process were generally similar across the 3 groups, with the exception of concerns related to changes in appearance: ∼50% of aesthetics receivers and naive considerers expressed worry around age-related changes in appearance compared with 28% of naive non-considerers.

**Table 2.** Concerns Related to Growing Older

Concern	Total *N* = 7588, %	Aesthetics receivers *n* = 3017, %	Naive considerers *n* = 2180, %	Naive non-considerers *n* = 2391, %
Change in my physical health	66	68	69	62
Losing mobility	55	57	52	57
Change in my mental health/losing memory	54	57	58	49
Change in my appearance/less attractive	45	53	54	28
Losing family/friends	44	44	46	42
Losing my independence	39	41	39	38
Being alone	39	39	44	34
Becoming invisible/perceive I am noticed less	29	32	34	20
My voice/perspectives are not valued as much	24	27	28	16
Being judged on what I wear/looking a certain a way	21	25	27	12
There are no concerns about getting older	8	5	3	16

Most respondents, regardless of their relationship with aesthetic medicine, perceived that there are many benefits to their subjective age (ie, the age someone *feels*) being lower than their chronological age. Respondents associated a lower subjective age with better physical (83%) and mental health (83%) as well as increased social engagement (82%). Furthermore, both aesthetics receivers (88%) and naive considerers (91%) believe that aesthetic treatments can help people achieve a lower subjective age; this sentiment was less prevalent, but not minimal, among naive non-considerers (62%).

**Figure 2.**
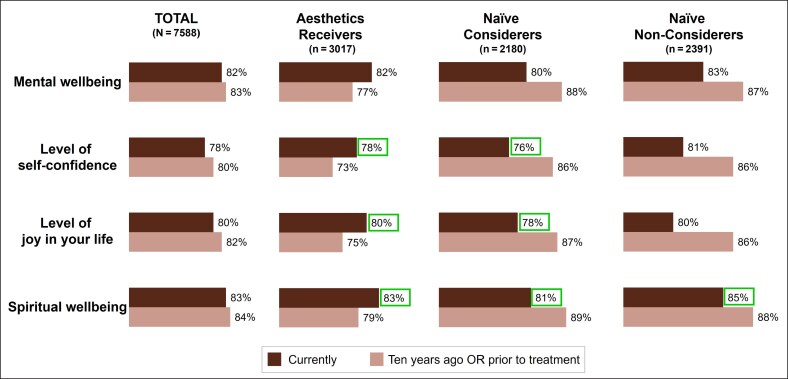
Psychological self-perceptions at time of survey and 10 years before survey. Respondents were asked to indicate their level of satisfaction with each aspect of their life currently and 10 years ago on a 6-point Likert scale (1 = extremely dissatisfied to 6 = extremely satisfied); satisfaction was defined as responses of “slightly satisfied,” “moderately satisfied,” or “extremely satisfied”. Note that the survey did not span 10 years; perceptions related to 10 years ago are retrospective and based on what the respondent perceived they felt 10 years before the survey. Percentages surrounded by boxes indicate significant differences from 10 years earlier (naive considerers and naive non-considerers) or before treatment (aesthetics receivers) as calculated with 95% CIs.

**Figure 3.**
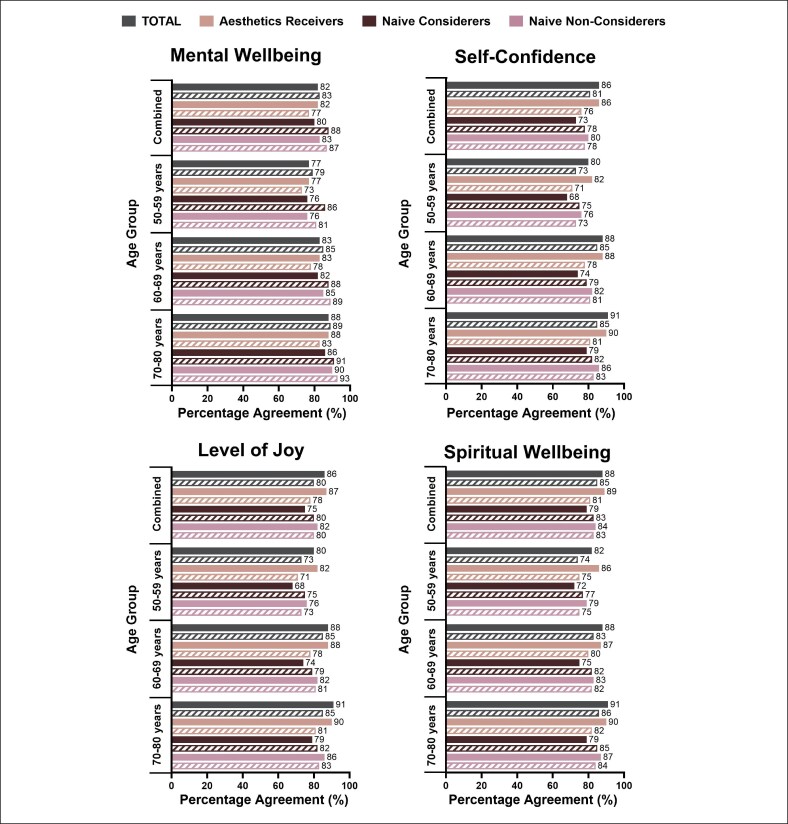
Psychological self-perceptions at time of survey and 10 years before survey based on age subgroup. Respondents were asked to indicate their level of satisfaction with each aspect of their life currently and 10 years ago on a 6-point Likert scale (1 = extremely dissatisfied to 6 = extremely satisfied); satisfaction was defined as responses of “slightly satisfied,” “moderately satisfied,” or “extremely satisfied”. Note that the survey did not span 10 years; perceptions related to 10 years ago are retrospective and based on what the respondent perceived they felt 10 years before the survey. Figure shows satisfaction with each psychological self-perception based on age subgroup and aesthetic engagement subgroup.

The majority of respondents across all groups reported being satisfied with their level of mental well-being (82%), self-confidence (78%), spiritual well-being (83%), and the level of joy in their lives (80%) at the time of the survey (Figure 2). When reflecting on their perceived satisfaction with these factors 10 years ago, respondents reported being slightly, but not significantly, more satisfied in the past. Satisfaction with these psychological self-perceptions did differ based on age, with the younger respondents (50-59 years) expressing generally less satisfaction with all 4 self-perceptions than either older subgroup (60-69 or 70-80 years) (Figure 3).

### Expectations for Health and Appearance While Aging

**Figure 4.**
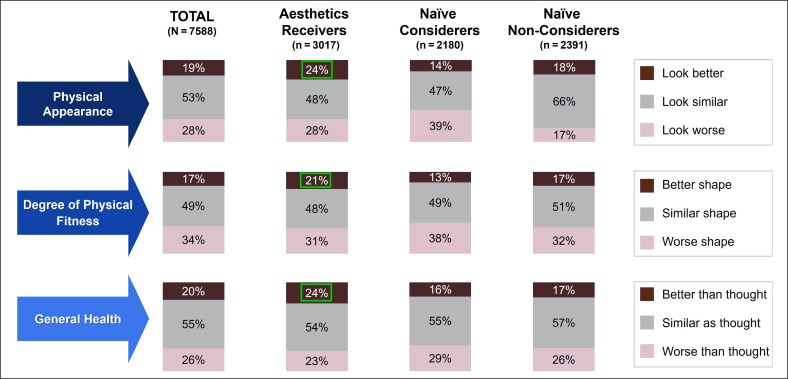
Expectations related to appearance and health 10 years ago compared with the time of survey. Respondents were asked to think back 10 years ago about the expectations they had for their physical appearance, degree of physical fitness, and general health. They were then asked to describe how their expectations aligned with the current state of each factor using a 3-point scale (better, similar, or worse). Percentages surrounded by boxes indicate significant differences from naive considerers and naive non-considerers.

The majority of respondents (∼75%) reported that they expect to look and feel good as they get older, but significantly more so if they received (look good, 77%; feel good, 79%) or were considering (look good, 77%; feel good, 78%) aesthetic treatment compared with naive non-considerers (look good, 65%; feel good, 70%). Significantly more respondents who were currently engaged with or were interested in aesthetic medicine expressed that they were worried about getting older (aesthetics receivers, 79%; naive considerers, 87%) than naive non-considerers (55%). A small proportion of respondents agreed that their appearance (19%) and health (20%) were better than they had anticipated 10 years ago. However, consumers who received aesthetic treatments were significantly more likely than other groups to agree that their appearance, fitness level, and overall health were better than they thought they would be now compared with their expectations 10 years ago, although the most common response was that their expectations from the past matched their current state of appearance or health (Figure 4).

About half of respondents thought societal expectations of maintaining one's appearance during aging had substantially or somewhat increased compared with what was expected 30 years ago (eg, their parents' generation), although this relationship is driven by aesthetics receivers (61%) and considerers (58%) because only 38% of naive non-considerers felt that expectations had increased. Reasons for greater expectations to maintain appearance include more options (68%), more exposure to one's appearance on social media (56%) and how older (eg, 50-70s) celebrities look vs before (47%).

The majority of respondents (71%) agreed that they want to look like the best version of themselves as they age; this sentiment was strongest among naive considerers (81%), followed closely by aesthetics receivers (77%), then naive non-considerers (54%). More female respondents (76%) than male respondents (59%) agreed that they want to look like the best version of themselves with age.

### Relationship Between Physical Appearance and Psychological Factors

**Figure 5.**
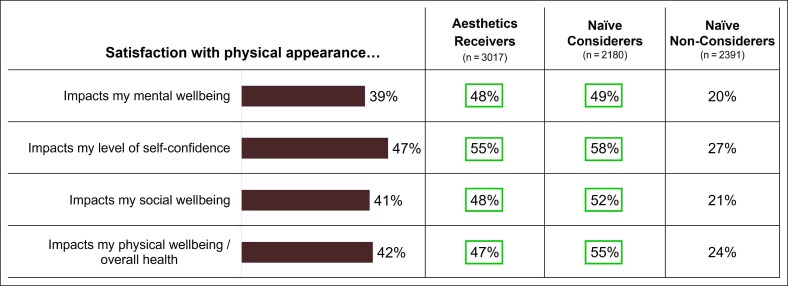
Association between satisfaction with physical appearance and psychological self-perceptions. Respondents were asked to assess the degree to which satisfaction with their physical appearance impacts psychological self-perceptions using a 5-point scale (1 = no impact, 2 = slight impact, 3 = moderate impact, 4 = strong impact, 5 = very strong impact). Data represent responses of “strong” or “very strong” impact. Percentages surrounded by boxes denote significant differences from naive non-considerers as calculated by 95% CIs.

Most aesthetics receivers and naive considerers were significantly more likely to agree that their perceived physical appearance impacts their mental and social well-being, particularly their self-confidence, whereas significantly fewer naive non-considerers linked physical appearance with mental or social well-being (Figure 5). Female respondents were more likely than male respondents to agree that physical appearance impacts well-being, with female naive considerers the most likely. In general, more concern with the aging process was associated with higher engagement with aesthetic medicine; significantly more aesthetics receivers (51%) and naive considerers (43%) expressed that they were “somewhat” or “definitely not” looking forward to aging compared with 40% of naive non-considerers.

### Perceptions of Aesthetic Treatments

The majority of all respondents (83%) agreed that there are emotional benefits associated with aesthetic treatments (Table 3). This association was strongest among aesthetics receivers (94%) and naive considerers (97%), whereas 57% of naive non-considerers agreed that aesthetic treatments had emotional benefits. The emotional benefits that respondents associated with aesthetic treatments included: increased self-confidence, feeling refreshed, to be the best version of themselves, and increased overall happiness. Perceived benefits of aesthetic treatments were similar across groups, although overall a smaller proportion of naive non-considerers expressed agreement with any listed potential emotional benefit. Additionally, the youngest age subgroup (50-59 years) tended to have less agreement that there were emotional benefits associated with aesthetic treatments than older surveyed respondents (Supplemental Figure 1).

**Table 3.** Emotional Benefits Associated With Aesthetic Treatment for Full Survey Population

Emotional benefit	Total *N* = 7588, %	Aesthetics receivers *n* = 3017, %	Naive considerers *n* = 2180, %	Naive non-considerers *n* = 2391, %
Increased self-confidence/self-esteem	47	53	55	33
To feel refreshed	38	44	45	23
Helps me be the best version of me	36	45	43	20
Puts me in a better mood in general	34	40	39	21
Want my appearance to match how I feel on the inside	33	39	40	20
Become happier	33	36	43	21
Allows me to worry less about my appearance	33	39	36	23
Feeling older—aesthetic treatment can help me feel younger	33	41	35	22
Perceive it is time to start focusing on me/self-care	29	33	38	15
Improvement in overall mental well-being (ability to pay attention, think clearly, memory)	25	29	31	16
Makes me more optimistic on things	25	29	33	15
I will feel seen	16	19	19	10
Job is stressful, but do not want to show that stress on my face	14	16	17	8
None—do not expect/receive any emotional benefit	17	6	3	43

The majority of aesthetics receivers (63%) and naive considerers (68%) expressed regret over not having received aesthetic treatments earlier in life, whereas 20% of naive non-considerers shared this regret. The primary reason that respondents expressed a wish to have engaged with aesthetic medicine earlier in life was that they would have received the benefits sooner. Naive non-considerers, however, reported that earlier engagement would have meant needing less treatment currently to improve their appearance (47%) than a desire to receive benefits of treatment sooner (29%).

## DISCUSSION

This multi-country online survey of adults aged 50 to 80 years provides key insights into this previously underrepresented population's perceptions of aging, as well as how these perceptions influence their relationship with aesthetic medicine. Results showed that satisfaction with psychological self-perceptions was generally quite high (∼80%), regardless of respondents' engagement with aesthetic medicine. There was strong agreement between all 3 groups that there are benefits to aging (eg, more time for hobbies/leisure, more time to spend with friends and family, retirement) as well as potential challenges (eg, changes in mobility, losing independence, losing memory or other changes in mental health). Importantly, the survey findings also suggest that the outlook that older adults have on aging impacts their relationship with aesthetic medicine, with a negative outlook (eg, worrying about getting older) being associated with engagement with aesthetic treatments.

Respondents' relationship to aesthetic medicine did influence some views on aging and aesthetic treatments. Naive non-considerers had an overall more accepting attitude toward aging than other groups, expressing less worry over getting older. Although the majority of respondents agreed that there is greater societal pressure today than 30 years ago to maintain a certain appearance as one age, only about one-third of naive non-considerers shared this sentiment. This may be driven by differences in perspective about the link between physical appearance and emotional well-being and may be influenced by naive non-considerers being less affected by external and societal factors. The present survey sought to explore perceptions around the aging process and did not capture information regarding the current state of respondent's physical or mental health (eg, diet, exercise regimen, presence or absence of diseases or disorders), nor did it delineate reasons why respondents might be, or not be, worried about aging, such as physical health, lifestyle, socioeconomic status or occupation, cognitive decline, changes in appearance, etc. As a result, it is not clear from the present data whether the older adults expressing little worry over aging engage in lifestyles that promote longevity, are simply accepting of the aging process, or whether their outlook on aging is influenced by other factors. Regardless, aesthetics receivers and naive considerers emphasized wanting to look like the best version of themselves as well as viewing aesthetic treatments as a means to accomplish this. Naive non-considerers were less likely to report wanting to look like the best version of themselves, which may point to appearance being less relevant to this group as a success factor associated with aging.

The majority of respondents, regardless of their engagement with aesthetic medicine, expressed high levels of satisfaction with multiple psychological self-perceptions, including their mental well-being and self-confidence. Among naive considerers, satisfaction with these self-perceptions generally declined with age, whereas aesthetics receivers had improved levels of satisfaction with these self-perceptions at the time of the survey compared with what they remembered from 10 years earlier. These findings are notable, as the high baseline levels of self-confidence and the similarity across subgroups may suggest that a lack of self-confidence is *not* a primary motivator for older adults to seek aesthetic treatment. This contrasts past literature that reported a desire to increase self-confidence as a key reason for adults aged 18 to 85 seeking both surgical and minimally invasive aesthetic treatments, although motivations were not broken down by age.^20^ Instead, older adults may view these treatments as a way to boost confidence by empowering patients in the aging process and supporting them to align self-image with external appearance. In line with this hypothesis about the role of aesthetic treatments as a tool for empowerment and greater agency during aging, aesthetics receivers and naive considerers expressed greater concern surrounding the aging process as well as stronger agreement that aesthetic treatments offer emotional benefits.

Among the naive non-considerers, satisfaction with psychological self-perceptions was high and generally maintained over time. These findings may suggest that factors *other* than appearance more strongly influence psychological self-perceptions such as mental well-being and self-confidence for non-considerers. This is supported by the finding that naive non-considerers were also the group least likely to link physical appearance to psychological self-perceptions. Naive non-considerers also may not be aware of potential psychological and psychosocial benefits of aesthetic treatments. Furthermore, as there is a weaker link between appearance and psychological factors among naive non-considerers, there may also be negative perceptions or biases related to the appearance-focused terms “aesthetic” or “cosmetic” as labels for the field and associated treatments that dissuade these older adults from engaging with aesthetic medicine. It is possible that alternative descriptors (eg, “wellness,” “longevity”) might pique interest for these treatments, but future research is needed to test this hypothesis. Alternatively, as fewer naive non-considerers reported that they expect to look and feel good as they get older compared with either other subgroup, naive non-considerers may not necessarily be uninterested in aesthetic procedures but may feel that it is too late for them to alter the course of their aging process.

It is important to note that the age distribution of the respondents was not even across the 3 eligible decades but was skewed toward the younger ages (ie, 41% of respondents were aged 50-59 years compared with 22% aged 70-80 years). The greater representation of the younger respondents may have a moderating or smoothing effect on the overall results and may distort the views of the oldest respondents, who are already facing more evident changes associated with advanced age. Indeed, the respondents aged 50 to 59 years tended to have slightly lower satisfaction with psychological self-perceptions than older respondents, regardless of their engagement with aesthetic medicine, as well as lower agreement that aesthetic treatments impart emotional benefits. This may suggest that this younger group has a more pragmatic view on aesthetic treatments, potentially because they are not yet facing the same psychological and physical challenges associated with aging. Furthermore, these findings suggest that expectations related to aesthetic treatments evolve with age even into later years, and greater psychological benefits may be associated with aesthetic treatments as individuals age. Although the present findings provide critical insight into the perceptions of aging and aesthetic medicine among a previously underrepresented population of older adults, future research is needed to better elucidate how these views may evolve decade by decade.

This survey study does have limitations worth mentioning. Although efforts were made to minimize selection bias (ie, sourcing from a general panel of consumers rather than an aesthetics-focused consumer group; predefining enrollment targets for aesthetic experience subgroups, age subgroups, gender, and country), selection bias may still have been present and may have contributed to the slightly lower numbers of naive considerers and naive non-considerers compared with aesthetics receivers. Another limitation is the self-reported nature of responses, particularly regarding past perceptions (eg, “How satisfied were you with each aspect of your life 10 years ago?”). Responses to these questions may be biased by poor recollection or interpretation of past experiences. Additionally, as with all survey studies, the direction of causality between factors cannot be ascertained. For example, the survey findings suggest that negative perceptions of aging are associated with increased engagement with aesthetic medicine, but it is unknown whether individuals who are more concerned about aging seek treatment with greater frequency or whether receiving aesthetic treatments influences their perception of aging. Another potential limitation is that the survey included only predetermined answer choices and did not include any free-response options for respondents to provide additional information or clarify answers. For example, 20% of respondents who were not interested in engaging with aesthetic medicine expressed regret over not having previously received aesthetic treatment; follow-up questions to ascertain the source of this regret only provided 2 answer options, thus limiting our understanding of factors (eg, cost, feeling as if it was too late) contributing to the somewhat high level of regret. The survey was also somewhat geographically limited by only including respondents from 8 countries; although the large sample size and geographic distribution of respondents increases the generalizability of the findings, there are likely regional and/or cultural differences influencing perceptions of aging that were not captured or explored in the present dataset. Future research and publications are warranted to understand ways in which aging may be viewed across cultures. Additionally, although the results suggest that a negative outlook on the aging process is associated with greater engagement with aesthetic medicine, the survey could not parse out if treatment supports positive psychological aging among these individuals (ie, their satisfaction with psychological self-perceptions is higher than it would be if they did not receive aesthetic treatment) or whether their engagement with aesthetic medicine is a reflection of their negative outlook on growing older. Regardless, the survey findings do suggest that these respondents receive positive emotional and psychological benefits from engaging with aesthetic medicine.

Future research and publications aim to explore generational, gender, and geographical/cultural differences in adults' outlook on the aging process and motivations for seeking aesthetic treatment. For example, whereas older adults may be seeking aesthetic treatments as a tool to *maintain* their existing appearance, younger generations may seek to *augment* or *change* their appearance (eg, lip filler for fuller lips among Gen Z patients) to improve confidence. As another example, male respondents were more likely than female respondents to report that they were not looking forward to growing older, but placed less importance on looking like the best version of themselves. This may suggest that older men are concerned more about functional changes than appearance-based changes during the aging process and additional research is warranted. Qualitative research, including interviews, would also allow for a deeper understanding of perceptions around aging and the role of aesthetic treatments. Future research could help to further characterize the concept of alignment between self-image and external appearance. Additionally, with rapid advancements in the regenerative and biostimulatory capabilities of aesthetic treatments and a shift toward a more therapeutic approach in aesthetic medicine, it will be important in the future to explore how evolutions within the field influence perceptions of aging and engagement with aesthetic treatments among older adults.

## CONCLUSIONS

Aging may be viewed as a privilege not afforded to everyone. It has been suggested that aging comes with unique perspectives, insights, and wisdom. Studying the psychology of aging and perceptions toward aesthetic treatments in older adults aged 50 to 80 years facilitates a deeper understanding of this patient population. Overall, the findings from this geographically diverse survey show varied attitudes toward aging among adults aged 50 to 80 years and highlight, for the first time, how these perspectives influence older adults' relationship with aesthetic medicine. For many older adults, aesthetic medicine can be a tool to enhance psychological self-perceptions and help one feel like the best version of themselves. For others, the aging process can be embraced without aesthetic medicine; it is possible that their interest may change in the future, but this survey shows that aesthetic treatment is sought by those who derive psychological benefit from physical improvements.

## Supplemental Material

This article contains supplemental material located online at https://doi.org/10.1093/asjof/ojaf127.

## Supplementary Material

ojaf127_Supplementary_Data
