# Supplementary material for: Perceptions About Aging and Aesthetics: A Global Study of Adults Aged 50 to 80 Years
Source: Aesthet Surg J Open Forum. 2025 Nov 13;7:ojaf127. doi: 10.1093/asjof/ojaf127 (PMC12663535; doi:10.1093/asjof/ojaf127)
Supplement: ojaf127_Supplementary_Data [file ojaf127_supplementary_data.docx]

**Supplemental Figure 1. Emotional benefits associated with aesthetic treatment based on age subgroup.** Respondents were asked “What are the main emotional benefits that you achieved (or want to achieve) with aesthetic treatments?” Data represent the percentage of respondents in each age subgroup agreeing with each of the provided potential emotional benefits. Panel (A) shows the total survey population without separating by aesthetic engagement, (B) shows the aesthetics receivers (n = 3017), (C) shows the naïve considerers (n = 2180), and (D) shows the naïve non-considerers (n = 2391).

**
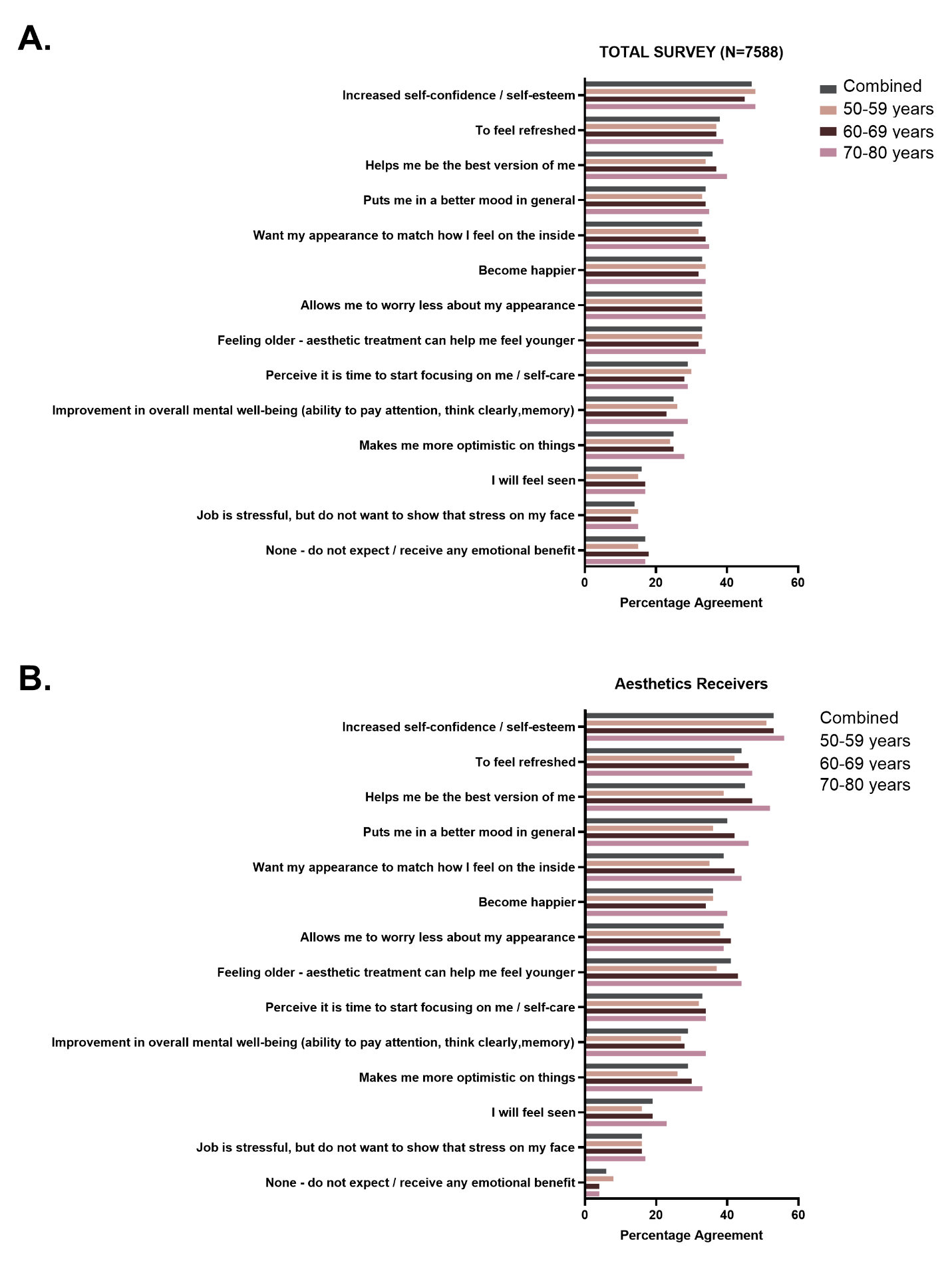
**

**
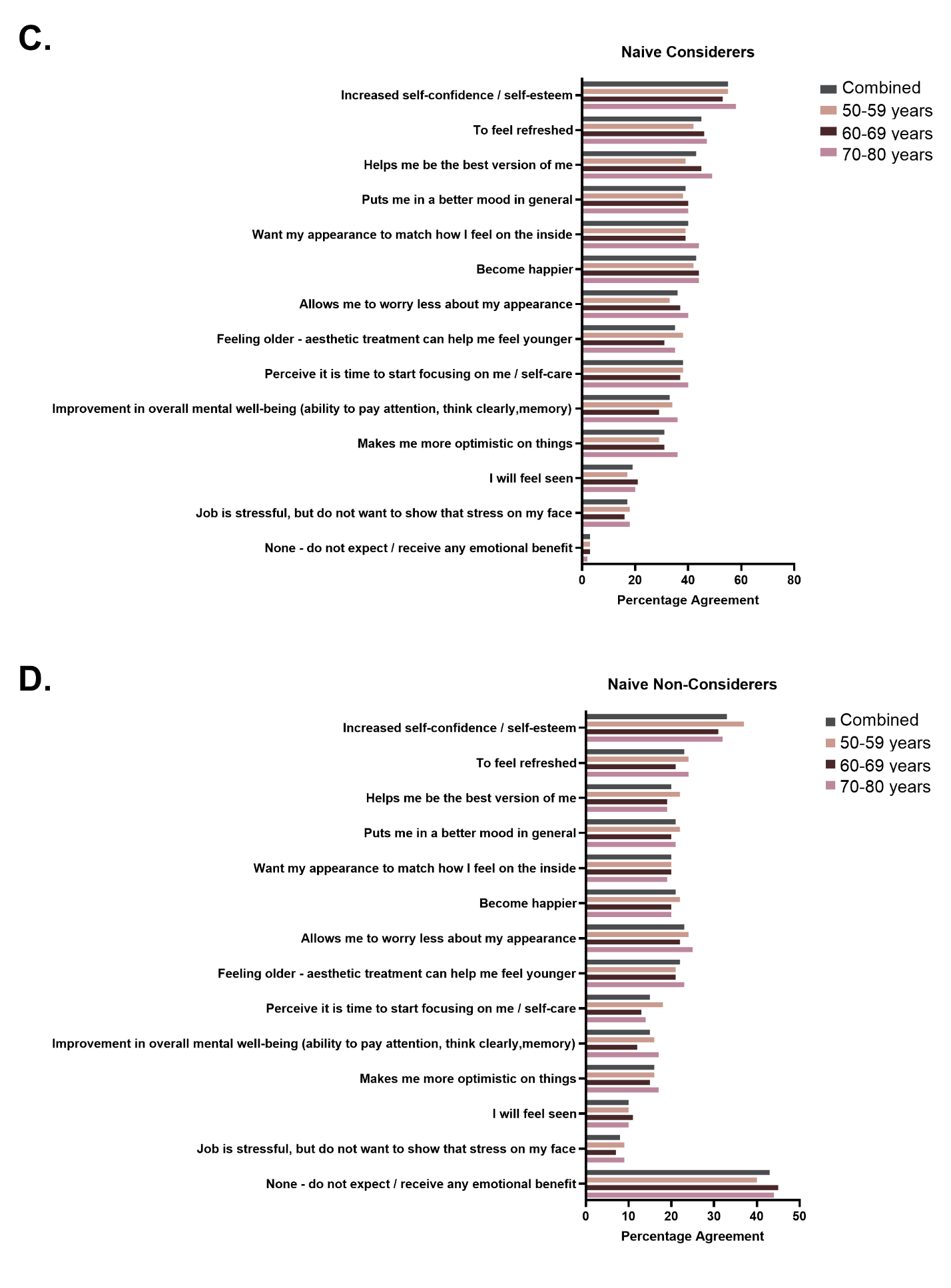
**
